# Disproportional signal of pericarditis with biological diseasemodifying antirheumatic drugs (bDMARDs) in patients with ankylosing spondylitis: a disproportionality analysis in the FAERS database

**DOI:** 10.3389/fphar.2024.1275814

**Published:** 2024-01-25

**Authors:** Shuang Xia, Yun-Fei Li, Emanuel Raschi, Bi-Kui Zhang, Yoshihiro Noguchi, Mayur Sarangdhar, Miao Yan, Jin-An Ma

**Affiliations:** ^1^ Department of Pharmacy, The Second Xiangya Hospital, Central South University, Changsha, China; ^2^ International Research Center for Precision Medicine, Transformative Technology and Software Services, Changsha, China; ^3^ Toxicology Counseling Center of Hunan Province, Changsha, China; ^4^ Department of Oncology, The Second Xiangya Hospital of Central South University, Changsha, China; ^5^ Pharmacology Unit, Department of Medical and Surgical Sciences, Alma Mater Studiorum-University of Bologna, Bologna, Italy; ^6^ Laboratory of Clinical Pharmacy, Gifu Pharmaceutical University, Gifu, Japan; ^7^ Division of Biomedical Informatics, Cincinnati Children’s Hospital Medical Center, Cincinnati, OH, United States; ^8^ Division of Oncology, Cincinnati Children’s Hospital Medical Center, Cincinnati, OH, United States; ^9^ Department of Pediatrics, University of Cincinnati College of Medicine, Cincinnati, OH, United States

**Keywords:** tumor necrosis factor inhibitors, interleukin-17 inhibitor, pericarditis, ankylosing spondylitis, pharmacovigilance, FDA Adverse Event Reporting system, disproportionality analysis

## Abstract

**Objective:** This study aimed to investigate the potential association between biological disease-modifying antirheumatic drugs (bDMARDs) and pericarditis and uncover relevant clinical characteristics in ankylosing spondylitis (AS).

**Methods:** Reports of pericarditis recorded in the FDA Adverse Event Reporting System (FAERS) (January 2004–December 2022) were identified through the preferred term “pericarditis.” Demographic and clinical characteristics were described, and disproportionality signals were assessed through the reporting odds ratio (ROR) and information component (IC). A significant signal was detected if the lower bound of IC (IC_025_) was more than zero.

**Results:** We found 1,874 reports of pericarditis with bDMARDs (11.3% of cases with fatal outcomes). Adalimumab (IC_025_ 3.24), infliximab (IC_025_ 4.90), golimumab (IC_025_ 5.40), certolizumab (IC_025_ 5.43), etanercept (IC_025_ 3.24), secukinumab (IC_025_ 3.97), and ustekinumab (IC_025_ 7.61) exhibit significant disproportionality signals compared to other medications in the FAERS database. After excluding pre-existing diseases and co-treated drugs that may increase the susceptibility of pericarditis, the disproportionality signal associated with infliximab, certolizumab, etanercept, secukinumab, and ustekinumab remained strong. Pericarditis cases associated with all bDMARDs were predominantly recorded in women aged 25–65 years.

**Conclusion:** More reports of pericarditis were detected with AS patients on bDMARDs than with other drugs in the overall database. Further studies are warranted to investigate the underlying mechanisms and identify patient-related susceptibility factors, thus supporting timely diagnosis and safe(r) prescribing of bDMARDs.

## 1 Introduction

Ankylosing spondylitis (AS) is a rare chronic inflammatory disorder that in its worst form can result in the bony fusion of vertebral joints, ultimately resulting in chronic back pain. In the previous decade, AS has become a recognized subgroup of the more comprehensive and prevalent diagnostic entity referred to as axial spondyloarthritis (Taurog et al., 2016). Non-steroidal anti-inflammatory drugs (NSAIDs) represent the first-line symptomatic treatment for pain and stiffness (Ortolan et al., 2023). The introduction of tumor necrosis factor inhibitors (TNFi), as the first biological disease-modifying antirheumatic drugs (bDMARDs) in axial spondyloarthritis, opened a new era in the management of this disease (Webers et al., 2023). In patients with active AS who failed to respond to conventional NSAID treatment, treatment with TNFi, including adalimumab, infliximab, golimumab, certolizumab, and etanercept, results in an excellent response (Sieper and Poddubnyy, 2017). Interleukin-17 inhibitors (IL-17i), such as secukinumab, ixekizumab, and brodalumab, are another type of bDMARDs for AS. The latest 2022 ASAS-EULAR recommendations (Ramiro et al., 2023) supported the use of TNFi, IL-17i, and targeted synthetic DMARDs (i.e., JAKi), depending on patient’s characteristics such as extra-musculoskeletal manifestations. Of note, cardiovascular risk management represents a key priority in patients with AS since various cardiovascular complications may occur (Atzeni et al., 2020). The cardiovascular safety of TNFi is still a matter of debate. While a number of studies have reported a reduction in sub-clinical atherosclerosis in AS as a consequence of their anti-inflammatory effect (Atzeni et al., 2020), a recent review (Hussain et al., 2021) showed that TNFi may increase the risk of adverse cardiovascular events (CVEs) because of stronger inhibition effects on TNFR2 (a cardioprotective receptor) than TNFR1 (an apoptotic receptor).

Pericarditis refers to the inflammation of the pericardial layers and is the most common form of pericardial disease. It may be associated with pericardial effusion that can result in impaired cardiac filling (tamponade) (Chiabrando et al., 2020). Drug-induced pericarditis, though uncommon, is a potentially life-threatening condition since it could result in pericardial tamponade, which could be fatal (Harnett et al., 2014). Previous case reports (Vyas et al., 2020; Lee et al., 2023; Obeidat et al., 2023) showed that pericarditis could be associated with TNFi in psoriatic arthritis. However, there are no reports of pericarditis with bDMARDs in AS. Moreover, pleuro-pericarditis was recently identified as a post-marketing safety signal for TNFi based on 42 well-documented cases reported in the international spontaneous reporting system WHO VigiBase (Zhang and Yue, 2020).

Therefore, large-scale pharmacovigilance archives are particularly suited to detect rare adverse events that may escape detection from clinical trials and may provide a comprehensive overview of unpublished case reports collected so far (Noguchi et al., 2021). This pharmacovigilance study used the FDA Adverse Event Reporting System (FAERS) to investigate the potential association between bDMARDs approved in AS and pericarditis.

## 2 Materials and methods

### 2.1 Study design and data source

The study was designed as a retrospective pharmacovigilance analysis of the FAERS database, which contains adverse event reports, reports of medication errors, and complaints about the quality of products that led to adverse events submitted to the FDA by healthcare professionals, consumers, and manufacturers. AERS*Mine* is a multi-cohort analyzing application designed to mine FAERS data across millions of patient reports (2004–2022, currently 19,089,556) (Sarangdhar et al., 2016). We performed a disproportionality analysis of pericarditis cases with bDMARDs in AS using data from the FAERS database. Ethical approval and patient consent were not needed because this study used de-identified data.

### 2.2 Definition of drugs, exposure, and cases of interest

We included nine bDMARDs: adalimumab, infliximab, golimumab, certolizumab, etanercept and secukinumab, ixekizumab, ustekinumab, and brodalumab. Exposure assessment considered these drugs recorded as suspects (“primary suspect” and “secondary suspect”) or concomitants. Only reports where AS was specified as a therapeutic indication were retained.

Adverse events in FAERS were coded through the so-called Medical Dictionary for Regulatory Activities (MedDRA) terminology (version 26.0) in terms of Preferred Terms (PTs), identifying unique signs and symptoms. To obtain a comprehensive understanding, we firstly detected the signals of all PTs within “noninfectious myocarditis/pericarditis” (Standardized MedDRA Query, SMQ) with bDMARDs. Then we focused on the signal of “Pericarditis” with bDMARDs. We only included pericarditis reports with a number more than five. For each pericarditis report, the following data were gathered: report year, reporter type (e.g., healthcare professionals and consumers), demographic information (gender and age), drugs, outcomes (with a focus on serious cases, i.e., those resulting in death, hospitalization or life-threatening condition, or disability), and co-reported adverse events.

### 2.3 Disproportionality analysis

To determine if pericarditis was differently reported with bDMARDs compared to other drugs in the FAERS, we performed the so-called case/non-case design. If the proportion of adverse events of interest is higher in patients exposed to a particular drug (cases) than in patients not exposed to this drug (non-cases), a disproportionality signal is generated (Faillie, 2019). Two different disproportionate measures were calculated, namely, the frequentist reporting odds ratios (RORs) and the Bayesian information components (ICs) to decrease the possibility of false-positive results. Significant disproportionality was noted when the lower limit of the 95% confidence interval of ROR (ROR_025_) >1 (Rothman et al., 2004) or the lower limit of the 95% confidence interval of IC (IC_025_) >0 (Bate et al., 1998). First, we used other drugs in the FAERS database as a comparator. To reduce the indication bias, we did not include NSAIDs as comparators in this study because a previous paper showed that NSAIDs could be used to treat pericarditis (Imazio et al., 2015). In addition, NSAIDs are usually used to treat stable AS. However, bDMARDs are usually used to treat more active AS. Therefore, NSAIDs are not suitable to be comparators in this study. We checked the website of Spondylitis Association of America (https://spondylitis.org/about-spondylitis/treatment-information/medications/) and the “2019 Update of the American College of Rheumatology/Spondylitis Association of America/Spondyloarthritis Research and Treatment Network Recommendations for the Treatment of AS and Nonradiographic Axial Spondyloarthritis” (Ward et al., 2019) and included the following medications as the second comparators: sulfasalazine, mesalazine (the active moiety of sulfasalazine) (Dekker-Saeys et al., 2000), methotrexate, tofacitinib, and upadacitinib. These agents are also utilized to treat more active AS.

A time trend analysis was also performed to explore the stability of the disproportionality signals over time. Moreover, we conducted the following sensitivity analyses to account for potential confounders like underlying comorbidities and bias:

(a) We excluded cases where pre-existing diseases (including “pericarditis,” “epstein-barr viral infections,” “cytomegalovirus infection,” “human herpesvirus 6 infection,” “parvovirus b19 infection,” “echovirus test positive,” “*mycobacterium tuberculosis* complex test positive,” “*borrelia burgdorferi* serology positive,” “*coxiella* infections,” “histoplasma infections,” “blastomyces infections,” “*candida* infections,” “toxoplasma infections,” “systemic lupus erythematosus,” “sjogren&apos_s syndrome,” “rheumatoid arthritis,” “scleroderma,” “eosinophilic granulomatosis with polyangiitis,” “familial Mediterranean fever,” “tumor necrosis factor receptor-associated periodic syndrome,” “sarcoidosis,” “inflammatory bowel disease,” “pericardial mesothelioma malignant” “lymphomas nec,” “non-small-cell lung cancer,” “small cell lung cancer,” “breast cancer,” “uremia,” “anorexia nervosa,” “pericardial disorders,” “postpericardiotomy syndrome,” “coronary artery bypass,” “cardiac pacemaker insertion,” “radiofrequency ablation,” “transcatheter aortic valve implantation,” “percutaneous coronary intervention,” “hypothyroidism,” “tuberculosis,” and “noninfectious pericarditis”) were recorded as possible alternative causes of pericarditis (Imazio et al., 2015; Chiabrando et al., 2020).
(b) We also excluded cases with co-treated drugs (including “procainamide,” “hydralazine,” “methyldopa,” “isoniazid,” “phenytoin,” “beta-lactam antibacterials, penicillins,” “doxorubicin,” “daunorubicin,” “fluorouracil,” “cyclophosphamide,” “minocycline,” “sulfasalazine,” “enalapril,” “clofarabine,” “carfilzomib,” “bortezomib,” “dasatinib,” “ceritinib,” “clozapine,” “nivolumab,” “pembrolizumab,” and “atezolizumab” (“ipilimumab” AND “nivolumab”: “sulfamethoxazole and trimethoprim” “interferon” “minoxidil” “lisinopril” “apixaban” “rivaroxaban” “streptokinase” “bromocriptine” “dantrolene”)] that may induce pericarditis (Imazio et al., 2015; Ma et al., 2021; Dababneh and Siddique, 2023) to minimize the so-called competition bias.
(c) We limited the reports from healthcare professionals and role code as “primary or secondary suspect,” respectively.

All these analyses were carried out to further test the robustness of disproportionality signals and enhance relevant clinical transferability.

Finally, subgroup analysis was conducted to investigate the influence of age and gender on disproportionate signals. In line with recent research on immune-related adverse events in patients receiving immune checkpoint inhibitors (Yang et al., 2023), we used IC delta to investigate the effect of age and gender. IC delta was calculated based on the observed-to-expected ratio between older adults (or males) and younger adults (or females) and was used to contrast the risk between the two groups. A positive (negative) IC delta was taken to reflect over (under-) reporting in one group if significant at the 5% significance level.

### 2.4 Drug interaction analysis

Considering that the co-administration of other drugs with bDMARDs may affect the disproportionate signals of pericarditis, we conducted drug interaction analysis using the Ω shrinkage to measure the drug–drug interactions because a previous study (Noguchi et al., 2020) showed that it is the most conservative among multiple algorithms. The detection criterion is the lower limit of the 95% CI of Ω (Ω_025_) > 0.

## 3 Results

### 3.1 Descriptive analysis

We detected that pericarditis was the most frequent PT term with bDMARDs within the noninfectious myocarditis/pericarditis (SMQ) (Table 1). From Q1, 2004 to Q4, 2022, we detected 281, 361, 269, 270, 248, 208, and 237 pericarditis cases with adalimumab, infliximab, golimumab, certolizumab, etanercept, secukinumab, and ustekinumab, respectively (Table 2). Most cases were reported from 2019 to 2022. Almost 34.0% (637/1874 cases) of pericarditis cases were reported by healthcare professionals. In the majority of the cases, bDMARDs were marked as primary suspects (179 cases) and secondary suspects (1584 cases). Except for two pericarditis cases with adalimumab in the elderly (age >65), other cases were recorded in adults (aged 25–65). A total of 11.3% (212/1874 cases) of pericarditis cases had fatal outcomes, while 18.2% (342/1874) cases needed hospitalization. Edema peripheral (930 cases), dizziness (948 cases), rheumatic fever (468 cases), dyspnea (383 cases), peripheral swelling (373 cases), and tachycardia (100 cases) were the most frequently co-reported adverse events.

**TABLE 1:** Disproportionality signals for noninfectious myocarditis/pericarditis (SMQ) with bDMARDs.

Category	Overall bDMARDs	Full database	ROR_025_	IC_025_
Total number of ICSRs available	109,943	19,089,556	—	—
Pericarditis	1,874	13,816	26.22	4.47
Red blood cell sedimentation rate increased	1,329	12,858	19.01	4.07
Pericardial effusion	154	24,144	0.95	−0.12
Extrasystoles	59	7,817	1.02	−0.05
Cardiac failure acute	42	6,414	0.84	−0.33
Myocarditis	45	9,430	0.62	−0.76
Electrocardiogram abnormal	33	9,082	0.45	−1.24
Atrioventricular block	22	8,146	0.31	−1.79
Bundle branch block left	35	5,242	0.83	−0.35
Dilatation ventricular	19	3,479	0.60	−0.85

bDMARDs, biological disease-modifying antirheumatic drugs; FAERS, FDA Adverse Event Report System; IC_025_, the lower limit of the 95% credibility interval of the information component; ICSR, individual case safety report; ROR_025_, the lower limit of the 95% credibility interval of the reporting odds ratio. When IC_025_ > 0 or ROR_025_ > 1, a significant safety signal was detected.

**TABLE 2:** Clinical features of pericarditis with bDMARDs.

Category/Drug	Adalimumab 281	Infliximab 361	Golimumab 269	Certolizumab 270	Etanercept 248	Secukinumab 208	Ustekinumab 237
Report year							
2004–2008	1 (0.4%)	29 (8.0%)	0 (0.0%)	0 (0.0%)	7 (2.8%)	0 (0.0%)	0 (0.0%)
2009–2013	3 (1.1%)	13 (3.6%)	1 (0.4%)	0 (0.0%)	5 (2.0%)	0 (0.0%)	0 (0.0%)
2014–2018	9 (3.2%)	9 (2.5%)	2 (0.7%)	2 (0.7%)	7 (2.8%)	5 (2.4%)	2 (0.8%)
2019–2022	268 (95.4%)	310 (85.9%)	266 (98.9%)	268 (99.3%)	229 (92.3%)	203 (97.6%)	235 (99.2%)
Reporter							
Healthcare professionals	89 (31.7%)	139 (38.5%)	84 (31.2%)	83 (30.7%)	81 (32.7%)	80 (38.5%)	81 (34.2%)
Consumer	22 (7.8%)	29 (8.0%)	14 (5.2%)	17 (6.3%)	23 (9.3%)	23 (11.1%)	16 (6.8%)
Unspecified	170 (60.5%)	193 (53.5%)	171 (63.6%)	170 (63.0%)	144 (58.1%)	105 (50.5%)	140 (59.1%)
Role code							
Primary suspect drug	22 (7.8%)	78 (21.6%)	12 (4.5%)	13 (4.8%)	17 (6.9%)	37 (17.8%)	0 (0.0%)
Secondary suspect drug	244 (86.8%)	293 (81.2%)	244 (90.7%)	243 (90.0%)	195 (78.6%)	151 (72.6%)	214 (90.3%)
Concomitant	26 (9.3%)	49 (13.6%)	30 (11.2%)	28 (10.4%)	42 (16.9%)	55 (26.4%)	23 (9.7%)
Sex							
Male	8 (2.8%)	33 (9.1%)	1 (0.4%)	1 (0.4%)	7 (2.8%)	7 (3.4%)	0 (0.0%)
Female	238 (84.7%)	283 (78.4%)	232 (86.4%)	234 (86.7%)	215 (86.7%)	178 (85.6%)	206 (86.9%)
Missing	35 (12.5%)	45 (12.5%)	36 (13.4%)	35 (13.0%)	26 (10.5%)	23 (11.1%)	31 (13.1%)
Age							
0–24	0 (0.0%)	0 (0.0%)	0 (0.0%)	0 (0.0%)	0 (0.0%)	0 (0.0%)	0 (0.0%)
25–65	154 (54.8%)	208 (57.6%)	146 (54.3%)	148 (54.8%)	130 (52.4%)	167 (80.3%)	147 (62.0%)
>66	2 (0.7%)	0 (0.0%)	0 (0.0%)	0 (0.0%)	0 (0.0%)	0 (0.0%)	0 (0.0%)
Missing	125 (44.5%)	153 (42.4%)	123 (45.7%)	122 (45.2%)	118 (47.6%)	41 (19.7%)	90 (38.0%)
Outcome							
Congenital anomaly	5 (1.8%)	5 (1.4%)	5 (1.9%)	5 (1.9%)	5 (2.0%)	5 (2.4%)	5 (2.1%)
Death	32 (11.4%)	30 (8.3%)	30 (11.2%)	30 (11.1%)	29 (11.7%)	31 (14.9%)	30 (12.7%)
Disability	104 (37.0%)	104 (28.8%)	103 (38.3%)	103 (38.1%)	102 (41.1%)	49 (23.6%)	102 (43.0%)
Hospitalization	49 (17.4%)	96 (26.6%)	35 (13.0%)	37 (13.7%)	48 (19.4%)	40 (19.2%)	37 (15.6%)
Life-threatening	49 (17.4%)	54 (15.0%)	47 (17.5%)	47 (17.4%)	52 (21.0%)	34 (16.3%)	47 (19.8%)
Other serious illnesses	271 (96.4%)	317 (87.8%)	262 (97.4%)	265 (98.1%)	234 (94.4%)	203 (97.6%)	232 (97.9%)
Co-reported adverse events							
Cardiac disorders							
Edema peripheral	141 (17.9%)	141 (17.9%)	142 (18.0%)	141 (17.9%)	83 (10.5%)	141 (17.9%)	141 (59.5%)
Dizziness	143 (17.8%)	148 (18.4%)	143 (17.8%)	143 (17.8%)	85 (10.6%)	143 (17.8%)	143 (60.3%)
Rheumatic fever	83 (17.7%)	84 (17.9%)	84 (17.9%)	84 (17.9%)	84 (17.9%)	49 (10.5%)	0 (0.0%)
Dyspnea	70 (18.3%)	71 (18.5%)	70 (18.3%)	69 (18.0%)	73 (19.1%)	30 (7.8%)	0 (0.0%)
Peripheral swelling	61 (16.4%)	78 (20.9%)	62 (16.6%)	61 (16.4%)	74 (19.8%)	37 (9.9%)	0 (0.0%)
Tachycardia	17 (17.0%)	18 (18.0%)	16 (16.0%)	17 (17.0%)	16 (16.0%)	16 (16.0%)	0 (0.0%)
Vascular disorders							
Hypertension	89 (17.7%)	90 (17.9%)	90 (17.9%)	90 (17.9%)	90 (17.9%)	55 (10.9%)	0 (0.0%)
Contusion	68 (18.7%)	69 (19.0%)	69 (19.0%)	69 (19.0%)	69 (19.0%)	20 (5.5%)	68 (28.7%)
Hemorrhage	5 (6.2%)	25 (30.9%)	0 (0.0%)	5 (6.2%)	21 (25.9%)	25 (30.9%)	0 (0.0%)

bDMARDs, biological disease-modifying antirheumatic drugs.

### 3.2 Disproportionality analysis

**FIGURE 1:**
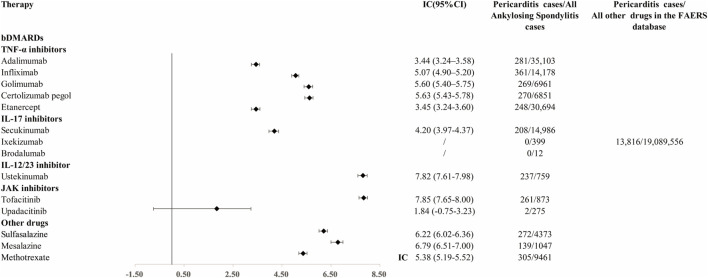
Disproportionality signal for pericarditis with TNFi, IL-17i, and IL-12/23 inhibitors and other medications for AS. IC, information component; 95% CI, 95% of credibility interval.

**FIGURE 2:**
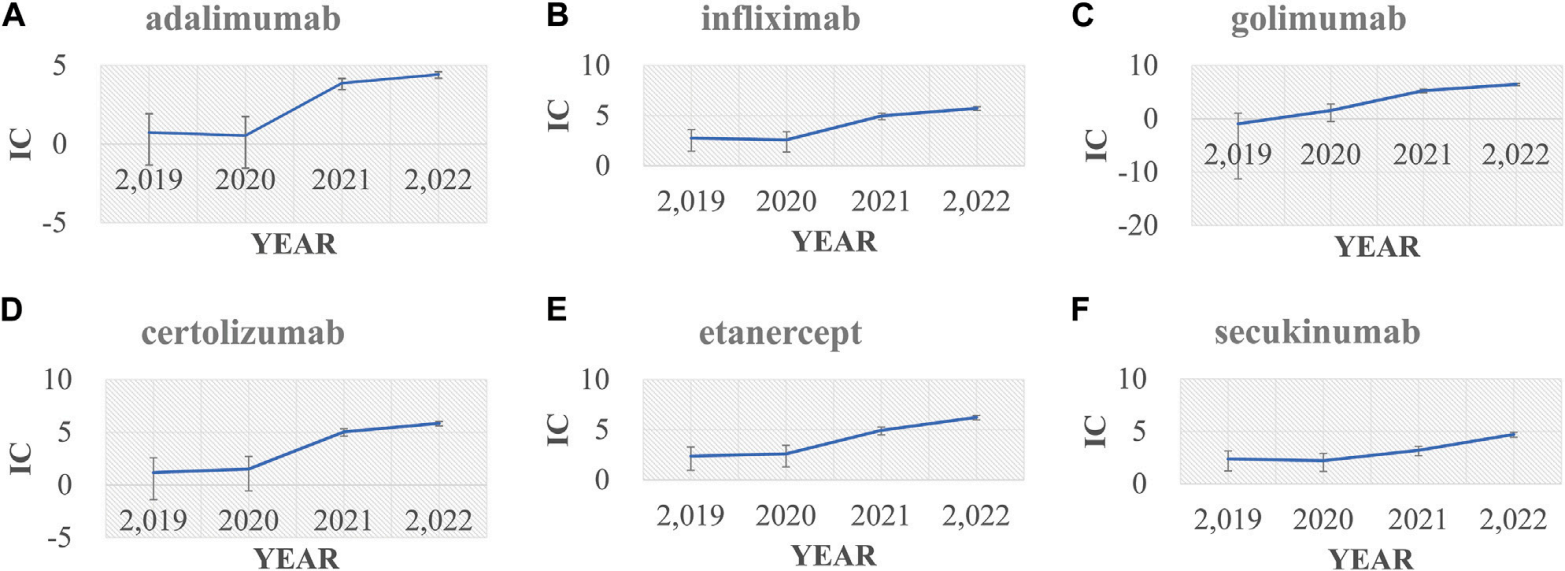
Information component (IC) and its 95% credibility interval over time for **(A)** adalimumab, **(B)** infliximab, **(C)** golimumab, **(D)** certolizumab, **(E)** etanercept, and **(F)** secukinumab. IC, information component.

In the primary analysis using all other drugs in the FAERS database as the comparator, all bDMARDs (adalimumab, infliximab, golimumab, certolizumab, etanercept, secukinumab, and ustekinumab) presented disproportionality signals. Although bDMARDs had lower disproportionality signals for pericarditis than tofacitinib, they presented higher signals of disproportionate reporting than other drugs in the FAERS database (Figure 1). The time trend analysis confirmed that six bDMARDs had a stable disproportionality signal of pericarditis from 2019 to 2022 (Figure 2). After accounting for drug-related competition bias, pre-existing disease, and a limited role code and report source in the sensitivity analysis, the pericarditis disproportionate signals with golimumab and adalimumab became weak. However, infliximab, certolizumab, etanercept, secukinumab, and ustekinumab still presented a higher disproportionate signal of pericarditis compared to tofacitinib, upadacitinib, and mesalazine (Table 3). By conducting subgroup analysis, we found that more pericarditis cases were reported in female patients receiving adalimumab, infliximab, golimumab, certolizumab, etanercept, and secukinumab (IC delta _975_ < 0, Supplementary File S1).

**TABLE 3:** Sensitivity analysis of pericarditis associated with drugs in AS compared with all other drugs in the FAERS database.

Drug	Corrected for drug-related competition bias, N, IC_025_	Corrected for suspect drugs and reports from healthcare professionals, N, IC_025_	Corrected for pre-existing disease, N, IC_025_	Signal consistency
Adalimumab	82/6499, 3.72	12/31655, −0.83	19/32436, −0.17	Weak (1/3)
Infliximab	130/7333, 4.30	85/12304, 3.83	96/13268, 3.78	Strong (3/3)
Golimumab	79/2674, 4.74	5/6012, −0.45	4/6392, −1.15	Weak (1/3)
Certolizumab	77/2090, 4.97	2/6050, −2.62	7/6097, 0.11	Intermediate (2/3)
Etanercept	71/17116, 2.18	37/28774, 1.32	44/28922, 1.45	Strong (3/3)
Secukinumab	62/3556, 4.01	40/13437, 2.47	46/14573, 2.45	Strong (3/3)
Ustekinumab	75/231, 6.47	0/355	5/461, 1.46	Intermediate (2/3)
Tofacitinib	70/206, 6.39	2/488, −0.68	2/387, −0.64	Weak (1/3)
Upadacitinib	1/55, −2.30	0/264	0/238	No signal (0/3)
Sulfasalazine	76/523, 6.11	97/3823, 5.42	9/3738, 1.17	Strong (3/3)
Mesalazine	53/108, 6.09	0/720	0/817	Weak (1/3)
Methotrexate	83/1460, 5.45	39/7181, 3.22	43/8237, 3.07	Strong (3/3)
Comparator	4773/7100723	4961/14603848	6170/16200491	—

Comparator in the sensitivity analysis is all other drugs in the FAERS database.

### 3.3 Drug interaction analysis

Our data showed that adalimumab (Ω_025_ = 5.5), infliximab (Ω_025_ = 6.2), golimumab (Ω_025_ = 7.8), certolizumab (Ω_025_ = 7.9), and ustekinumab (Ω_025_ = 2.0) had strong signals of drug interactions with tocilizumab (Supplementary File S2). Etanercept (Ω_025_ = 0.6) and secukinumab (Ω_025_ = 0.5) showed weak signals of drug interactions with tocilizumab (Supplementary File S3).

## 4 Discussion

To the best of our knowledge, this is the first study to investigate the association of pericarditis with bDMARDs for AS in the real-world setting of spontaneous reporting. By conducting a multimodal approach, comprising multiple stepwise disproportionality analyses, these findings contribute to the ongoing discussion on the cardiovascular risk of patients with AS and strengthen the hypothesis that bDMARDs as a class may increase patient’s susceptibility to pericarditis.

First, our disproportionality analysis detected that pericarditis was significantly reported with TNFi, IL-17i, and IL-12/23 inhibitors, with other drugs in the FAERS database as the comparators. Ustekinumab presented the highest disproportionate signal of pericarditis among all bDMARDs. There was only one case report (Tominaga et al., 2021) that showed that tuberculous pericarditis occurred with ustekinumab treatment for Crohn’s disease. Previous research (Rubin et al., 2022) reported that four cases of pericarditis were associated with tofacitinib, which is consistent with the strong disproportionate signal of pericarditis with tofacitinib in this study. However, our sensitivity analysis found that the disproportionate signal of pericarditis with ustekinumab and tofacitinib became weak, while infliximab, etanercept, and secukinumab still presented strong signals across three sensitivity analyses. Our study showed that the reports of pericarditis with bDMARDs have rapidly increased since 2019. We checked the data from EudraVigilance and found a similar tendency (Supplementary File S4). Previous research (Patone et al., 2022) showed that COVID-19 vaccination or SARS-CoV-2 infection may increase the risk of pericarditis in patients. Considering the similar timeline of a huge increase in pericarditis reports in bDMARDs and an outbreak of COVID-19, we investigated the influence of COVID-19 on the reports of pericarditis in our analysis. We combined tocilizumab, remdesivir, baricitinib, and other FDA-approved COVID-19 vaccines with bDMARDs and searched for pericarditis reports (limit the indication as AS) in the FAERS database. There is only one report of pericarditis with the combination of adalimumab and COVID-19 vaccines. No reports of pericarditis were detected for the co-administration with bDMARDs and remdesivir or baricitinib. However, we detected hundreds of pericarditis reports with the combination of tocilizumab and bDMARDs. We further limited COVID-19 as the indication and tocilizumab as the drug of interest, and no reports of pericarditis were detected in the FAERS database. This indicated a weak influence of COVID-19 itself on the signals of pericarditis with bDMARDs. Previous literature (Shikama et al., 2001; Imai et al., 2022) showed that pericarditis may be associated with IL-6 receptor antagonists in POEMS syndrome and eosinophilic granulomatosis with polyangiitis. We further investigated if the co-administration of bDMARDs and tocilizumab would increase the reports of pericarditis in AS. Our data showed that the co-administration of tocilizumab with bDMARDs may synergistically increase the reports of pericarditis in patients with AS. Tocilizumab is an FDA-approved agent for COVID-19. Its utilization during COVID-19 may contribute to the increased reports of bDMARDs in AS. More research is warranted to further investigate the mechanisms of drug interactions.

Second, this study depicted the clinical characteristics of pericarditis with bDMARDs. Pericarditis was found to predominantly occur in women compared to men. This is consistent with previous research (Zhang and Yue, 2020). Moreover, another review (Stovall et al., 2022) indicated that axial spondyloarthritis has equal prevalence in women and men. This study indicated that women may be more susceptible to pericarditis than men when they receive DMARDs. Previous pharmacovigilance studies also showed that pleuro-pericarditis was more commonly reported in women (Zhang and Yue, 2020). Another research (Rusman et al., 2018) showed that women seemed to be more prone to infections during TNFi treatment than men. Infection may further induce pericarditis (Imazio et al., 2010). Increased infection risk in women receiving TNFi may contribute to the increased pericarditis reports. Moreover, Vermeire et al. (2003) showed that antinuclear antibodies were associated with the female sex (odds ratio, 3.166; 95% confidence interval, 1.167–8.585; *p* = 0.024) in patients on anti-tumor necrosis factor treatment for Crohn’s disease. Previous research (Goswami et al., 2018) showed that pericarditis was diagnosed in women, with 25.4% (104/409) of 409 patients with systemic lupus erythematosus. A higher risk of pericarditis in women was found for cutaneous lupus erythematosus (Olbrich et al., 2023). Those literature works indicated that anti-tumor necrosis factor treatment may increase the risk of lupus in women. In addition, pericarditis is a common cardiac manifestation of lupus. Further research is warranted to provide more evidence regarding this issue.

Third, our study supports the reporting association of pericarditis with TNFi and IL-17i in AS. A recent high impact review (Braun et al., 2017) summarized cardiovascular comorbidity in inflammatory rheumatological conditions. In this review, we found no evidence of pericarditis as an AS comorbidity. By the way, we found that there were only four pericarditis reports with an event date early compared to the start date of bDMARDs (one for etanercept, one for adalimumab, and two for infliximab), which was a very small percentage among all pericarditis reports with bDMARDs. The time-to-onset data further supported that pericarditis occurred after the administration of bDMARDs for AS (Supplementary File S5). These sources of evidence support that pericarditis may not be a complication of AS. This study only focuses on investigating the potential association of pericarditis with bDMARDs in AS but not in rheumatoid arthritis or psoriatic arthritis because previous studies (Ogdie et al., 2015; Sparks et al., 2019) reported an increased incidence of major adverse cardiovascular events in rheumatoid arthritis, psoriatic arthritis, or psoriasis. This was confirmed by a recent pooled genome-wide association study (Wang et al., 2023), which showed that rheumatoid arthritis itself increases the risk of heart failure. However, previous research (Fang et al., 2022) showed that JAKi and TNFi in rheumatoid arthritis have comparable safety issues and mortality rates. Another meta-analysis (Cai et al., 2023) showed that targeted therapies did not show a higher occurrence of all CVEs in PsO/PsA (RR = 1.03; 95% CI 0.74–1.43l; *p* = .85) compared to the placebo. Our findings showed that bDMARDs, especially infliximab, etanercept, and secukinumab, significantly presented a disproportionate signal of pericarditis compared to other medications for AS after accounting for confounding factors. Regarding the possible mechanism of pericarditis with bDMARDs, we believe that there are three possible aspects (direct cardiotoxicity, infection induced pericarditis, lupus-induced pericarditis). To begin with, TNFi may directly induce cardiotoxicity and increase the risk of pericarditis. A previous review (Hussain et al., 2021) showed that greater inhibition of TNFR2 (a cardioprotective receptor) than TNFR1(an apoptotic receptor) results in cardiovascular morbidity associated with TNFi. In addition, previous research showed that patients with inflammatory joint diseases initiating bDMARDs treatment had four times increased risk of serious infection compared with the general population (Krabbe et al., 2021). In addition, infection may further induce pericarditis. Lastly, recent research (De Bandt et al., 2005; Costa et al., 2008; Moulis et al., 2014) showed that TNFi had a higher potential to induce lupus than other drugs. As previously discussed, pericarditis may be a manifestation of systemic lupus erythematosus, cutaneous lupus erythematosus, and drug-induced lupus. This showed that pericarditis could be caused by bDMARD-related systemic lupus erythematosus. Much research is warranted to unveil the mechanism of pericarditis with bDMARDs.

This study has some limitations. First, the incidence of pericarditis following TNFi or IL-17i cannot be determined because the number of patients exposed to the drugs is unknown. Second, the source of reports in the FAERS database is heterogeneous, including non-health professionals, such as consumers and lawyers. However, we corrected this in our sensitivity analysis. Third, the detailed diagnosis information including radiography, biochemistry test, disease activity and duration, and the level of antinuclear antibodies is absent in the FAERS database, which may introduce confounding factors in our analysis. Fourth, this study does not allow us to infer causality (ROR and IC are not risk measures). Moreover, the contribution of additional drugs and AS itself to the underlying risk of pericarditis cannot be excluded. A recent study (Micallef et al., 2023) showed that a high number of adverse drug reaction reports for COVID-19 vaccines in EudraVigilance have the potential to affect routine statistical signal detection activities, which may also have an influence on the signal detection of this study.

Nonetheless, our study has some important strengths (Bihan et al., 2020). By using one of the largest publicly accessible pharmacovigilance databases, FAERS, this study contributed to the cumulative knowledge about the potential reporting association of pericarditis with bDMARDs for AS in an unselected real-world population, an evolving clinical issue. Moreover, we conducted stepwise sensitivity analyses to evaluate the robustness of results, accounting for potential co-reporting biases and using sulfasalazine, mesalazine (the active moiety of sulfasalazine), methotrexate, tofacitinib, and upadacitinib as comparators, which could provide a clinical perspective. More studies are warranted to validate this finding.

## 5 Conclusion

In patients with active AS, we found an increased reporting of pericarditis with bDMARDs, including TNFi and IL-17i, notably when compared with other medications in the FAERS database. Pericarditis could be potentially considered among the spectrum of major adverse cardiovascular events with bDMARDs in patients with AS. Further investigations are needed to better elucidate patients’ susceptibility (e.g., female sex) and the potential underlying autoimmune mechanism. The remarkable proportion of serious cases further calls clinicians to increase awareness of this life-threatening safety issue; this will finally support early detection and safe(r) prescribing of bDMARDs in AS patients.

## Data Availability

The original contributions presented in the study are included in the article/Supplementary Material; further inquiries can be directed to the corresponding author.
