# Supplementary material for: Disproportional signal of pericarditis with biological diseasemodifying antirheumatic drugs (bDMARDs) in patients with ankylosing spondylitis: a disproportionality analysis in the FAERS database
Source: Front Pharmacol. 2024 Jan 25;15:1275814. doi: 10.3389/fphar.2024.1275814 (PMC10850349; doi:10.3389/fphar.2024.1275814)
Supplement: Supplementary file 3 [file Presentation1.PPTX]

## Slide 1
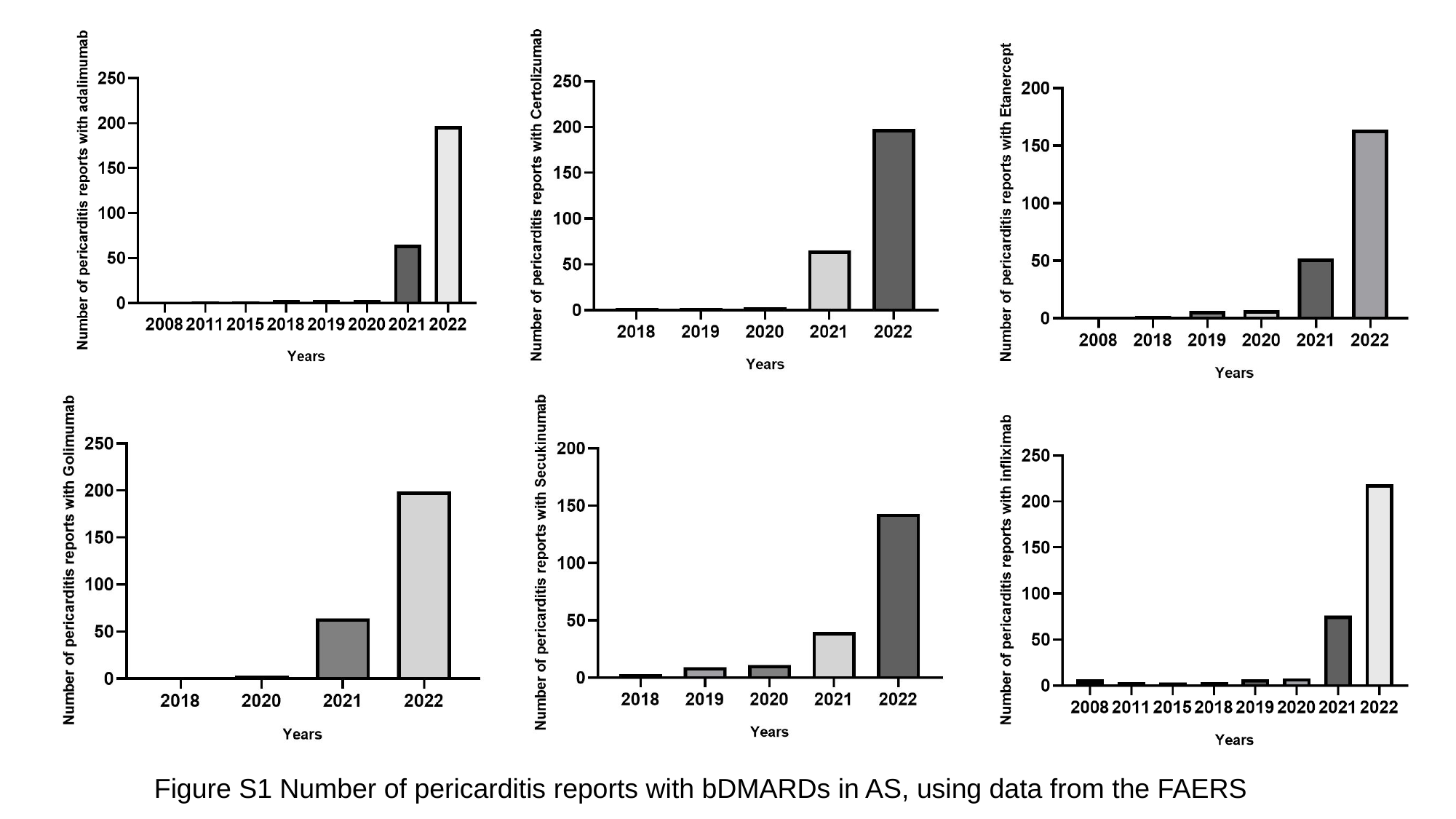

Figure S1 Number of pericarditis reports with bDMARDs in AS, using data from the FAERS

## Slide 2
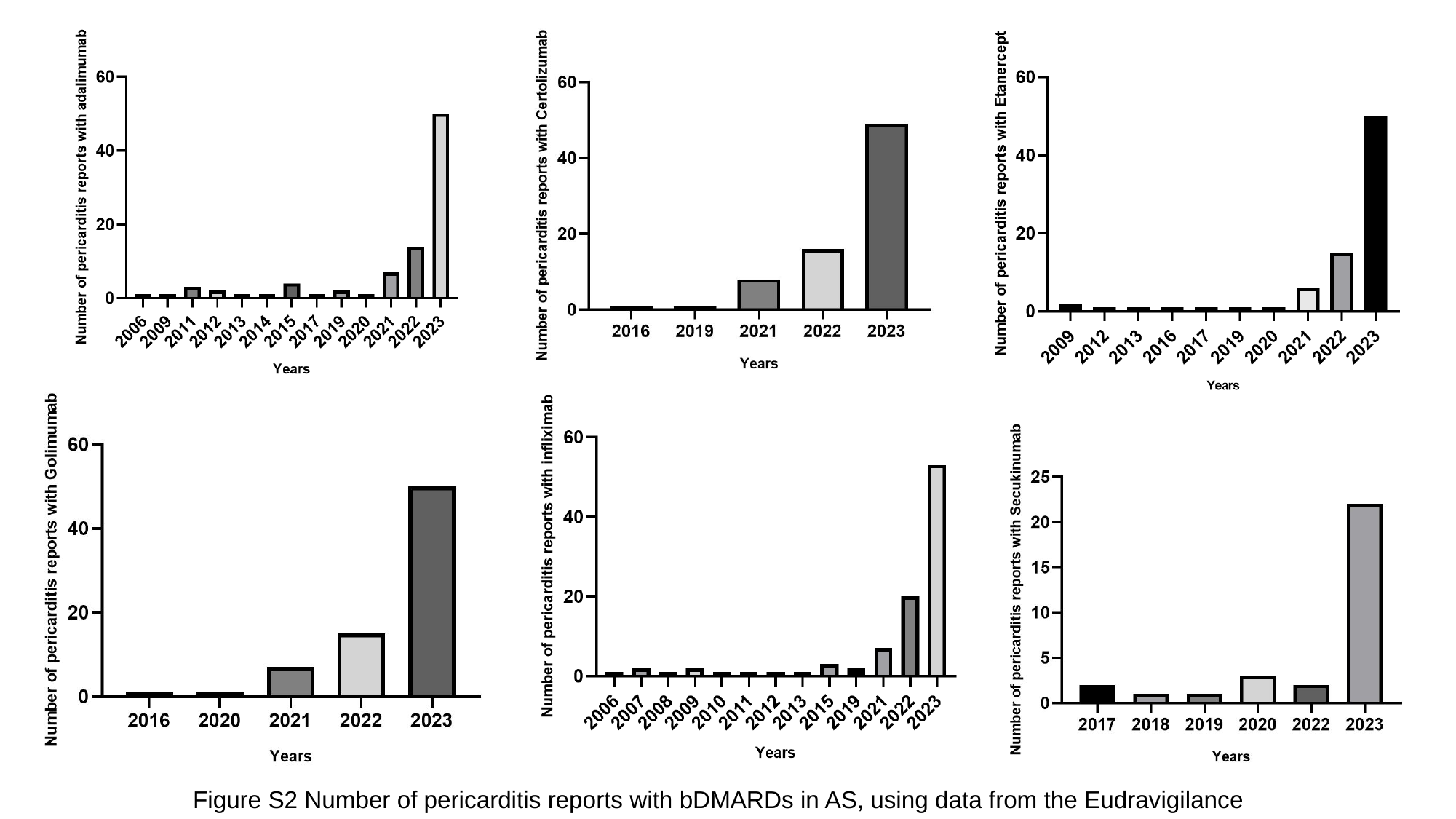

Figure S2 Number of pericarditis reports with bDMARDs in AS, using data from the Eudravigilance
